# Nephroprotective Properties of Antidiabetic Drugs

**DOI:** 10.3390/jcm12103377

**Published:** 2023-05-10

**Authors:** Christian Gerdes, Nicolle Müller, Gunter Wolf, Martin Busch

**Affiliations:** Department of Internal Medicine III, University Hospital Jena, D-07747 Jena, Germany

**Keywords:** chronic kidney disease, diabetes mellitus, treatment, nephroprotection, SGLT2 inhibitors, GLP1 receptor agonists, antidiabetic drugs, eGFR, albuminuria

## Abstract

Chronic kidney disease (CKD) is associated with increased morbidity and mortality, especially from cardiovascular (CV) causes, and especially in people with diabetes mellitus (DM). Already the presence of DM increases CV risk and potentiates the risk of CKD. Therefore, besides glycemic control, prevention and treatment of CKD to slow its progression are of clinical importance. A significant nephroprotective effect of novel antidiabetic drugs, namely sodium-glucose cotransporter 2 inhibitors (SGLT2-I) and glucagon-like peptide 1 receptor agonists (GLP1-RA), has been shown on top of their glucose-lowering effects and was confirmed in cardiovascular outcome trials. GLP1-RA mainly reduced the risk of macroalbuminuria, whereas SGLT2-I were also associated with a lower risk of declining glomerular filtration rate (GFR) over time. The nephroprotective effects of SGLT2-I are also evident in people without DM. According to current guidelines, SGLT2-I and/or GLP1-RA are recommended for people with DM who have chronic kidney disease and/or increased cardiovascular risk. However, other antidiabetic drugs offer nephroprotective properties, which will also be discussed in this review.

## 1. Introduction

Chronic kidney disease (CKD) is associated with increased morbidity and mortality [1], especially from cardiovascular (CV) causes [2]. People with CKD and diabetes mellitus (DM) are particularly affected [3], as the presence of DM doubles CV risk in the general population [4]. Already, the presence of micro- or macroalbuminuria in individuals with DM increases the risk of CV mortality, which is highest if the serum creatinine increases to the extent that the glomerular filtration rate (GFR) declines [5]. On the other hand, DM potentiates the risk of CKD, including end-stage kidney failure [6]. Another fact is the coincidence of several CV risk factors in people with CKD, such as the presence of hypertension, hyperlipidemia, older age, or the tendency to be obese and/or to have DM [7]. All factors promote CV morbidity and the progression of CKD.

Therefore, in individuals with CKD and DM, besides glycemic control, slowing the progression of CKD, especially to reduce CV risk, should be a goal. Moreover, well-described CV risk factors should be managed. Ultimately, a reduction in CV events should be achieved, a feature antidiabetic drugs are lacking until 2015 [8]. However, starting with sodium glucose transporter 2 inhibitors (SGLT2-I), more recently developed anti-diabetic drugs show promising results in protection from CV events, besides their potential to reduce the progression of CKD in individuals with DM. Recently, SGLT2-I demonstrated nephroprotective properties in people without DM, too.

In this review, we focus mainly on the nephroprotective properties of SGLT2-I and glucagon-like peptide 1 receptor agonists (GLP1-RA), both through the description of pathophysiologic mechanisms and clinical results. However, in this regard, we also want to look back at well-established antidiabetics such as insulin, metformin, sulfonylureas, and dipeptidyl peptidase-4 inhibitors (DPP4-I).

Therefore, we searched MEDLINE, Google Scholar, and the Cochrane Central Register of Controlled Trials with a focus on phase 3 randomized controlled trials.

## 2. Insulin

In people with type 2 DM, subcutaneous insulin therapy can be necessary throughout the course of the disease. In the UKPDS trial, intensive glucose-lowering therapy compared to conventional therapy showed a reduction in progression to microalbuminuria after 9 (relative risk: 0.76; 99% CI 0.62–0.91) and 12 years (relative risk: 0.67; 99% CI 0.53–0.86) and a reduction in a doubling of plasma creatinine after 12 years (relative risk: 0.26; 99% CI 0.07–0.91), but no significant effect on the progression to end-stage kidney disease or renal death [9]. In the intensive glucose-lowering therapy arm, approximately 42.5% of patients were treated with insulin. In other trials, such as ACCORD, improved glycemic control (target HbA_1c_ < 6%) showed a reduction in the development of microalbuminuria (HR 0.81; 95% CI 0.70–0.94) and macroalbuminuria (HR 0.68; 95% CI 0.54–0.86), too [10]. Notably, the use of intensive therapy to target normal glycated hemoglobin levels increased mortality but did not significantly reduce major CV events. Therefore, intensive therapy was terminated early, and people continued standard therapy (target HbA_1c_ 7.0–7.9%) until the study ended [11]. At transition, the intensive therapy group had a higher risk of doubling serum creatinine or a more than 20 mL/min/1.73 m^2^ decrease in estimated GFR (HR: 1.07; 95% CI: 1.01–1.16), which might be induced by reduced glomerular hyperfiltration. However, the effect was not significant after the transition to the standard of care at the end of the study [10]. In ACCORD, the prescription of insulin was high in both therapy arms (intensive: 77.3%, standard: 55.4%). The incidence of hypoglycemia requiring medical or any other assistance was significantly higher in the intensive therapy arm [11]. Other trials, such as VADT and ADVANCE (see below), showed a consistent effect of intensive glycemic control regarding the reduction of albuminuria. However, the rates of CV events were unaltered [12,13].

Apart from the glucose-lowering potential and therefore improved glycemic control, insulin showed no convincing nephroprotective properties except for a certain drop in albuminuria induced by improved glycemic control [14].

## 3. Metformin

Metformin is an orally administered drug that reduces blood glucose levels in people with type 2 DM by inhibiting hepatic gluconeogenesis, slowing intestinal glucose absorption, and improving hepatic and muscular insulin sensitivity. In animal models, metformin showed pleiotropic nephroprotective and anti-fibrotic effects by reducing cellular stress, inducing autophagy, and inhibiting the production of reactive oxygen species and mediators of hypoxia-induced kidney injury [15]. Several vasoprotective effects might have a positive influence on microvascular changes as well and thus might contribute to renoprotective properties [16]. Unfortunately, evidence from randomized controlled clinical trials with primary renal endpoints investigating metformin in people with DM is missing. Retrospective data showed a reduced rate of renal function decline and death with metformin compared to sulfonylureas [17].

## 4. Sulfonylureas

Sulfonylureas are administered orally. These drugs enhance insulin secretion by inhibiting K_ATP_-channels in pancreatic β-cells. The resulting reduced potassium outflow in β-cells depolarizes cell membranes and activates voltage-gated calcium channels. Increased intracellular calcium levels promote insulin secretion [18]. Furthermore, sulfonylureas reduce glucagon secretion by α-cells [19] and enhance peripheral insulin sensitivity [20].

The ADVANCE-trial randomized 11,140 patients to receive either intensive glucose control, aiming for an HbA_1c_ of 6.5% or lower, achieved by gliclazide (and add-on therapy), or standard glucose control as recommended by local guidelines, achieved by other glucose-lowering medication. After a median of 5 years of follow-up, the incidence of new or worsening nephropathy was significantly reduced (HR 0.79, 95% CI 0.66–0.93), driven by a reduction in new-onset macroalbuminuria (HR 0.70, 95% CI 0.57–0.85). As the use of other sulfonylureas in the standard glucose group was high (57.1%), the ADVANCE-trial only gives indirect hints for a nephroprotective effect of sulfonylureas achieved by improved glycemic control. Furthermore, there were no significant effects on major CV events or deaths from CV causes [13].

Additionally, an AHRQ-review and a Cochrane-review found no significant reduction of renal endpoints when comparing sulfonylureas or the combination of metformin and sulfonylureas to other monotherapies or metformin-based combination therapies, respectively. However, sulfonylureas in combination with metformin showed an increased risk for weight gain compared to placebo and metformin (weight difference 3.4 kg, 95% CI 1.4–5.4) and an increased risk for hypoglycemia (relative risk 3.93, 95% CI 0.71–21.88) [21].

## 5. Dipeptidyl Peptidase-4 (DPP-4) Inhibitors

DPP-4 inhibitors showed anti-inflammatory and anti-fibrotic renal effects in animal models. These properties result partly from the accumulation of incretin hormones (e.g., GLP-1) and possibly from incretin-independent mechanisms, as DPP-4 is expressed in the kidney [22,23]. Furthermore, DPP-4 inhibitors stimulate natriuresis and diuresis through GLP-1-induced inhibition of sodium-hydrogen exchanger 3 (NHE3) in the proximal tubular system [24]. As angiotensin (AT) II stimulates NHE3 via AT II1-receptors, the combination of RAS- and DPP-4-inhibitors might be favorable.

DPP-4 inhibitors can be orally administered. DPP-4 inhibitors are a heterogenous class of drugs, and most evidence regarding renal effects is available for linagliptin [22]. Linagliptin possesses a high penetration rate into kidney tissue [25]. In mice, therapy with telmisartan and linagliptin reduced albuminuria and markers of oxidative stress [26]. Moreover, linagliptin, independently of GLP-1, induced stromal cell-derived factor 1α (SDF-1α), stimulating antifibrotic effects [27].

In clinical trials, DPP-4 inhibitors showed no reduction in renal or CV endpoints [28]. Nevertheless, the CAROLINA trial demonstrated CV noninferiority of linagliptin compared to glimepiride in people with type 2 DM [29]. Among adults with type 2 DM and high CV and renal risk, linagliptin showed no superiority regarding a composite CV- or kidney outcome (HR 1.04, 96% CI 0.89–1.22) compared to placebo [30]. However, a meta-analysis showed a reduced risk of new-onset albuminuria with linagliptin compared to placebo [31].

## 6. Glucagon-like Peptide-1 (GLP-1) Receptor Agonists (GLP-1-RA)

Apart from weight loss and glycemic control, GLP-1-RA demonstrated various nephroprotective properties. GLP-1-RA showed a reduction of oxidative stress markers (via inhibition of NADP(H) oxidase) beyond glucose-lowering [32,33]. In animal models, GLP-1 receptor agonism downregulated the expression of tubulointerstitial tumor necrosis factor alpha (TNFα), monocyte chemoattractant protein-1 (MCP-1), collagen I, and fibronectin [34,35]. Through inhibition of NHE3, localized in the proximal tubular cells, GLP-1-RA increased natriuresis [36]. Although effects on the reduction of declining GFR have not been shown, the risk of progression of albuminuria was reduced in clinical trials, most likely due to glucose-lowering and extra-glycemic effects [35]. GLP-1-RA is mostly applied subcutaneously, except for semaglutide, which can be given orally as well.

In a prespecified secondary analysis of the LEADER trial, liraglutide showed a reduction of a composite renal endpoint consisting of new-onset persistent macroalbuminuria, persistent doubling of serum creatinine level, end-stage kidney disease, or renal death, compared to placebo (HR 0.78, 95% CI 0.67 to 0.92). This result was primarily driven by a lower rate of new-onset persistent macroalbuminuria (HR 0.74, 95% CI 0.60 to 0.91) and independent of baseline renal risk (including reduced eGFR < 60 mL/min/1.73 m^2^ or microalbuminuria/macroalbuminuria) [37]. Unfortunately, an adjustment to account for differences in other risk factors, e.g., glycemic control, has not been made [38].

The SUSTAIN-6 trial showed a lower rate of worsening nephropathy (including persistent macroalbuminuria, persistent doubling of serum creatinine level, and a creatinine clearance of less than 45 mL/min/1.73 m^2^ or the need for chronic renal replacement therapy) with semaglutide compared to placebo (HR 0.64, 95% CI 0.46 to 0.88). Similar to liraglutide, this result was primarily driven by a lower rate of persistent macroalbuminuria (HR 0.54, 95% CI 0.37 to 0.77) [39]. Both LEADER and SUSTAIN-6 are CV outcome trials and were not powered to assess renal endpoints. Therefore, no differences in the need for renal replacement therapy or renal death were observed. Consequently, the ongoing FLOW trial is investigating the effect of semaglutide on a composite primary kidney outcome defined as persistent eGFR decline of ≤50%, end-stage renal disease, and renal or CV death in people with type 2 DM and impaired kidney function compared to placebo. The study completion date is anticipated for August 2024 and might elucidate the nephroprotective effect of GLP-1 receptor agonism.

The GRADE trial demonstrated more effectiveness in maintaining glycemic control with liraglutide (or glargine) compared to glimepiride or sitagliptin in people with type 2 DM receiving metformin [40]. Secondary analysis showed fewer cases of any CV event, mainly driven by hospitalization due to heart failure, with liraglutide compared to glimepiride or sitagliptin and benefits regarding blood pressure control compared to glargine, glimepiride, or sitagliptin. In the per-protocol analysis, liraglutide had a lower risk of new-onset moderate albuminuria than glimepiride or glargine. However, this effect was not seen in the intention-to-treat analysis [41].

Finally, compared to SGLT2-I, GLP1-RA showed comparable effects regarding new-onset persistent macroalbuminuria. However, a lower rate of worsening of eGFR, new-onset end-stage renal disease, or renal death was only seen with SGLT2-I [42].

## 7. Dual Glucose-Dependent Insulinotropic Peptide (GIP) and GLP-1-RA

Although GIP-receptors are not present in kidneys, anti-inflammatory effects mediated by GIP might be beneficial in people with DM and chronic kidney disease. In animal models, GIP-agonism is associated with decreased interleukin-6 levels and increased adiponectin levels, which are associated with reduced insulin resistance [43,44,45]. A post-hoc analysis of the SURPASS-4 trial showed a less pronounced reduction of eGFR with tirzepatide compared to insulin glargine (between-group difference 2.2 mL/min/1.73 m^2^, 95% CI 1.6 to 2.8) and a lower occurrence of a composite kidney endpoint (HR 0.58, 95% CI 0.43 to 0.80) [46]. Unfortunately, there were no adjustments for glycemic control and changes in body weight with tirzepatide compared to insulin glargine. The SURPASS-CVOT trial with a primary CV endpoint (myocardial infarction, stroke, or CV death) is currently ongoing, and study completion is anticipated in 2024.

## 8. Sodium-Glucose Cotransporter 2 (SGLT2)-Inhibitors

### 8.1. Nephroprotective Mechanisms of Sodium-Glucose Cotransproter 2 (SGLT2)-Inhibitors

**Figure 1:**
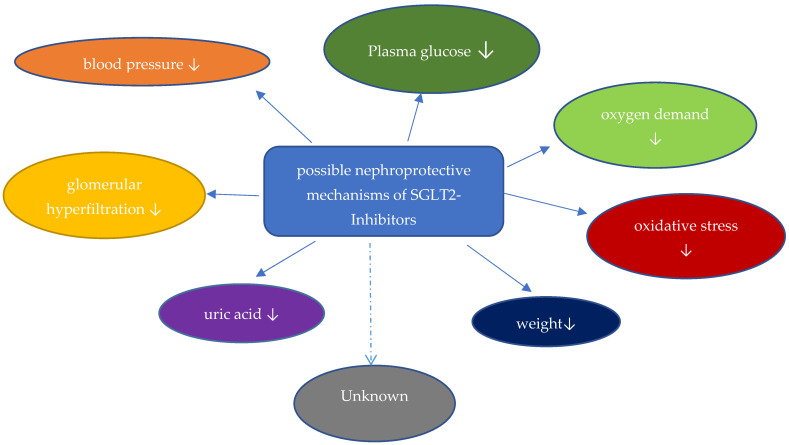
Nephroprotective mechanism of SGLT-2-inhibitors.

The kidney contributes to glucose homeostasis through endogenous glucose filtration, glucose reabsorption, glucose production, and glucose utilization. Approximately 160–180 g of glucose is filtrated and reabsorbed in normal glucose-tolerant individuals. SGLT2 is localized in the early proximal segment of the tubule and reabsorbs 80–90% of the filtrated glucose. SGLT1 is the primary transporter for glucose absorption in the intestine but is localized in the proximal tubule as well, where 10–20% of filtrated glucose is reabsorbed through SGLT1. The ratio of sodium to glucose cotransport is 1:1 for SGLT2 and 2:1 for SGLT1. The sodium-potassium ATPase on the basolateral membrane of proximal tubular cells actively moves sodium out of the cells, generating a sodium gradient for sodium-glucose cotransport from the tubular to the intracellular site via SGLT. Glucose is then, through GLUT 2 transporters, passively moved into the interstitial space. Therefore, inhibition of SGLT2 causes glucosuria and natriuresis. As the transport capacity of SGLT1 is maximized after inhibition of SGLT2, less than 50% of filtrated glucose is excreted in individuals on SGLT2-I, reducing the glucose-lowering potential. The following mechanisms are discussed as nephroprotective properties of SGLT2-I (Figure 1):

### 8.2. Glucose-Lowering Potential

Through inhibition of glucose reabsorption, urinary glucose excretion increases by approximately 50–80 g glucose/day, leading to lower fasting and postprandial plasma glucose levels. Depending on background therapy and baseline glycemic control, an HbA1_c_ reduction of 0.5–0.8% is achieved [47]. As plasma glucose levels decrease, nonenzymatic glycation and oxidation of proteins and lipids, and therefore, advanced glycation end-products (AGEs), are reduced. AGEs are associated with oxidative stress and inflammation [48]. Moreover, SGLT2-I ameliorates glucotoxicity by increasing ß-cell function and insulin sensitivity [49].

### 8.3. Blood Pressure Lowering

SGLT2 inhibition, through natriuresis and osmotic glucosuria, increases diuresis and, therefore, reduces extracellular fluid and plasma volume, causing improved blood pressure control [50]. Furthermore, weight loss of approximately 2.4% might ameliorate blood pressure. In clinical trials, SGLT2-I, compared to placebo, demonstrated a reduction of systolic and diastolic blood pressure of 3.77 mmHg and 1.75 mmHg, respectively [51]. SGLT2-I shows properties to stimulate the renin-angiotensin system (RAS) [52,53], so the combination of SGLT2-I with inhibitors of the renin-angiotensin system is suitable and was used in the vast majority of CV and renal end-point trials [54,55,56,57]. Finally, inhibition of cardiac sympathetic nerves by SGLT2-I is speculated [58].

### 8.4. Uric Acid

CKD and DM type 2 are associated with elevated serum uric acid levels. Conversely, hyperuricemia is associated with the onset and progression of CKD and CV mortality [59]. Increased glucosuria presenting on the GLUT 9 isoform 2, caused by SGLT2-inhibition, may inhibit uric acid reabsorption and therefore increase uric acid excretion [60]. Through SGLT2-I, serum uric acid concentrations decrease by approximately 0.3–0.9 mg/dL, potentially supporting the blood pressure-lowering properties of SGLT2-I [61].

### 8.5. Hemodynamic Considerations and Reduction of Albuminuria

Glomerular hyperfiltration, associated with increased intraglomerular pressure, is an important factor in the progression of CKD. Glomerular hyperfiltration stimulates glomerular hypertrophy, leading to glomerulosclerosis and progressive nephron loss. Finally, nephron loss itself is raising glomerular hyperfiltration in the remaining functional glomeruli, amplifying the process [50].

In DM, chronic and elevated glucosuria triggers the expression and increases the reabsorptive activity of SGLT2 in proximal tubular cells. Therefore, glucose and sodium reabsorption into tubular cells is increased, leading to tubular stress, e.g., through the production of AGEs, resulting in tubular hypertrophy. Furthermore, the intratubular sodium concentration of the distal tubule system, including the macula densa, is reduced. Hence, via tubuloglomerular feedback and intrarenal RAS-activity, the efferent arteriolar tone is raised. In addition, the decreased synthesis of ATP, which is then converted to adenosine by macula densa cells, causes reduced afferent arteriolar tone. Both afferent vasodilation and efferent vasoconstriction potentiate intraglomerular pressure and therefore trigger hyperfiltration [52,53,62], leading to glomerular hypertrophy and subsequently to glomerulosclerosis, progredient albuminuria, and nephron loss. Moreover, albuminuria and proteinuria trigger tubulointerstitial inflammation and profibrotic mechanisms [63].

Through SGLT2-inhibition, sodium delivery to the macula densa increases, restoring tubuloglomerular feedback. In young individuals with type 1 DM without RAS-inhibition, SGLT2-I reduced renal hyperperfusion by afferent vasoconstriction through increased adenosine levels [52]. In people with type 2 DM with RAS-inhibition, SGLT2-I reduced mainly efferent arteriolar tone [53]. Both afferent vasoconstriction and efferent vasodilation reduce intraglomerular pressure and hence ameliorate hyperfiltration [62,64].

Comparable to RAS-inhibition, SGLT2-I, by reducing hyperfiltration, induces an initial and reversible reduction in the estimated glomerular filtration rate, named the eGFR “dip”. In the EMPA-REG OUTCOME trial (see below), 28% of individuals with empagliflozin had an eGFR dip of >10%, while only 1.4% had a decline of >30%. Predictors of eGFR dipping are co-medication with diuretics and more advanced CKD [65]. Nevertheless, SGLT2-I slowed the decline of eGFR over time in comparison to placebo. Furthermore, through the reduction of intraglomerular pressure, albuminuria is reduced [62], contributing to the recovery of renal function. Presumably, approximately 30–40% of reductions in micro- and macroalbuminuria in trials with individuals with DM type 2 are induced by intrarenal hemodynamic effects [58].

### 8.6. Reduction of Podocyte Injury

In a mouse model, empagliflozin reduced mesangial expansion and increased podocyte autophagy, preventing podocyte detachment and loss, which then turned into a decline in albuminuria. By inducing fatty-acid oxidation, SGLT2-I might also reduce lipid content and lipotoxicity in podocytes, promoting their integrity [62].

### 8.7. Hypoxia and Hypoxia-Inducible Factors

The main driver of oxygen demand in the kidney is sodium reabsorption. In DM, proximal tubular glucose and sodium reabsorption by SGLT2-transporters is increased, resulting in a higher activity of the energy-consuming basolateral sodium-potassium-ATPase and, finally, an increased oxygen demand. Oxygen supply is primarily controlled by renal perfusion, which is impaired because of microvascular damage in DM. Therefore, renal hypoxia results from a mismatch of oxygen demand and supply, leading to hypoxia-induced nephron loss. Due to the hyperfiltration of the remaining nephrons, a vicious cycle is created [66].

Renal hypoxia and increased oxidative stress activate hypoxia-inducible factor 1α (HIF-1α) and suppress HIF-2α, promoting inflammation, glomerulosclerosis, and tubular fibrosis. As HIF-2α is activating erythropoietin synthesis in peritubular interstitial cells, renal hypoxia, and oxidative stress deteriorate renal anemia. SGLT2-I reduces renal oxygen demand and therefore lowers HIF-1α and promotes HIF-2α, stimulating erythropoiesis and thereby renal oxygen supply, besides reducing tubular fibrosis [67].

### 8.8. Aestivation

Aestivation (Latin “aestas”, meaning summer) is an evolutionarily conserved self-preservation strategy to enable physiological adaptation to water and/or energy shortages. SGLT2-I-induced glucosuria and natriuresis create energy and dehydration stress, triggering counterbalancing metabolic and physiological adaptations. Through the induction of enzyme cascades in liver and muscle cells, osmotic diuresis and energy expenditure are reduced. Therefore, energy utilization in the liver, kidney, and heart is optimized [68].

### 8.9. Renal Endpoints in Clinical Trials with SGLT2-Inhibitors

The reduction of CV- and renal endpoints is the primary goal of therapy in individuals with DM. Therefore, before approval, new anti-glycemic drugs need to show CV non-inferiority compared to placebo. In individuals with DM and CV disease or high CV risk, first-line therapy with SGLT2-I or, if contraindicated, GLP-1-receptor agonists is recommended [69].

Additionally, a reduction of CV endpoints and CV non-inferiority trials for SGLT2-I in people with DM at high CV risk showed a reduction of renal endpoints. Therefore, trials with primary renal endpoints were designed. Table 1 gives an overview of large clinical trials with primary renal or primary CV endpoints. SGLT-2-I is administered orally.

### 8.10. Empagliflozin

#### 8.10.1. Empagliflozin in Diabetes Mellitus

The EMPA-REG-OUTCOME trial randomized 7020 patients with type 2 DM and established CV disease at high risk for CV events to receive either 10 or 25 mg of empagliflozin or placebo. The mean eGFR of the pooled empagliflozin group (10 or 25 mg) at baseline was 74.1 mL/min/1.73 m^2^ and 25.9% of participants had an eGFR of <60 mL/min/1.73 m^2^. A prespecified secondary renal endpoint, consisting of progression to macroalbuminuria, doubling of serum creatinine (with a reduction of eGFR ≤ 45 mL/min), initiation of renal replacement therapy, or renal death, was significantly reduced in individuals receiving empagliflozin (HR 0.61, 95% CI 0.53 to 0.70). A post hoc assessment showed consistent effects regardless of baseline eGFR or albuminuria of the secondary renal endpoint, after exclusion of progression to macroalbuminuria [70].

#### 8.10.2. Empagliflozin in Heart Failure

The EMPEROR-Reduced trial randomized 3730 patients with chronic heart failure and an ejection fraction of 40% or less with (nearly 50%) or without DM to receive 10 mg of empagliflozin or placebo. Empagliflozin reduced the secondary endpoint decline in eGFR over the treatment period (difference: 1.73 mL/min/1.73 m^2^/year, 95% CI 1.10 to 2.37). The prespecified efficacy composite renal endpoint (chronic dialysis or renal transplantation or eGFR ≤ 15 mL/min/1.73 m^2^ or a fall in eGFR of ≥40%) was significantly reduced with empagliflozin, too (HR 0.50, 95% CI 0.32 to 0.77). Unfortunately, no subgroup analyses of individuals with and without DM are available [71].

In EMPEROR-Preserved, 5988 patients with chronic heart failure and an ejection fraction of 40% or more with or without DM (nearly 50% had DM) received 10 mg of empagliflozin or placebo. Comparable to EMPEROR-Reduced, the decline in eGFR over the treatment period was reduced in the empagliflozin group (difference: 1.36 mL/min/1.73 m^2^/year, 95% CI 1.06 to 1.66), although a composite renal endpoint showed no significant difference [72].

The EMPAG-HF trial demonstrated the effectiveness of the early addition of empagliflozin at a dose of 25 mg per day in acute decompensated heart failure to increase urine output. 43.3% of patients in the empagliflozin group and 34.5% of patients in the placebo group had type 2 DM. Markers of renal function or injury were not affected, but eGFR after 30 days was reduced in the placebo group compared to empagliflozin [61]. The EMPULSE trial confirmed the safety and effectiveness of empagliflozin in acute decompensated heart failure independently of baseline eGFR. Secondary analyses demonstrated no decline in eGFR after 90 days and lower rates of investigator-reported acute kidney injury, although not reaching statistical significance (placebo-group: 7.2%; empagliflozin-group: 3.8%; *p* = 0.0935) [73].

#### 8.10.3. Empagliflozin in Chronic Kidney Disease

Finally, the EMPA-KIDNEY trial randomized 6609 individuals with an eGFR between 20 and 45 mL/min/1.73 m^2^ or an eGFR between 45 and 90 mL/min/1.73 m^2^ and an additional urinary albumin-to-creatinine ratio (UACR) of at least 200 mg/g to receive either 10 mg of empagliflozin or placebo. Only 46% of individuals had DM. The mean eGFR at baseline was 37.3 mL/min/1.73 m^2^ and 34.5% of participants had an eGFR of <30 mL/min/1.73 m^2^. The mean UACR was only 412 (94–1190) mg/g [74]. Comparable to the previous trials, most patients (around 85%) were treated with RAS-inhibitors. At the time of the formal interim analysis, EMPA-KIDNEY was stopped early due to its significant efficacy. The primary endpoint, consisting of progression of kidney disease (defined as end-stage kidney disease (initiation of chronic dialysis or kidney transplantation or a sustained decrease in eGFR to less than 10 mL/min/1.73 m^2^), a sustained decrease of eGFR > 40%, or renal death), and death from CV causes, was significantly reduced in the empagliflozin group (HR 0.72, 95% CI 0.64 to 0.82) compared to placebo. With regard to secondary endpoints, empagliflozin significantly reduced hospitalization from any cause, but neither overall mortality nor the composite endpoint of hospitalization for heart failure (HHF) or CV death were significantly reduced. Prespecified subgroup analyses showed a consistent effect in individuals with or without DM and across a broad range of eGFR (20 to 90 mL/min/1.73 m^2^). In contrast to EMPA-REG-OUTCOME, subgroup analysis of patients with albuminuria < 30 mg/g showed no significant effect on the primary outcome, but the decline in eGFR was nevertheless reduced in these individuals [75].

### 8.11. Dapagliflozin

#### 8.11.1. Dapagliflozin in Diabetes Mellitus

The DECLARE-TIMI 58 trial in individuals with DM and established CV disease or multiple risk factors (mean eGFR 85.2 ml/min/1.73 m^2^, 7.4% with eGFR < 60 mL/min/1.73 m^2^) showed a reduction of a secondary composite renal-specific endpoint consisting of eGFR decline of >40% to less than 60 mL/min/1.73 m^2^, end-stage kidney disease, and renal or cardiovascular death (HR 0.53, 95% CI 0.43 to 0.66) with dapagliflozin compared to placebo. Subgroup analyses showed a consistent effect independent of albuminuria and baseline eGFR. As DECLARE-TIMI 58 met only one of its dual primary outcomes, these analyses should be considered hypothesis-generating [76].

#### 8.11.2. Dapagliflozin in Heart Failure

The DAPA-HF trial randomized 4744 patients with heart failure and an ejection fraction of <40% to receive either dapagliflozin or placebo. Only 42% of individuals had DM. A secondary composite renal endpoint (eGFR decline of >50%, end-stage kidney disease, death from kidney disease, or any cause) showed no significant difference between dapagliflozin and placebo over a median of 18.2 months [77]. The DELIVER trial analyzed patients with heart failure and an ejection fraction of >40%. A composite kidney endpoint (≥50% decline in eGFR, end-stage renal disease, or renal death) was not affected by treatment with dapagliflozin [78]. Admittedly, in both DAPA-HF and DELIVER, the incidence rate of the kidney composite outcome was low.

#### 8.11.3. Dapagliflozin in Chronic Kidney Disease

The DAPA-CKD trial randomized 4094 individuals with an eGFR of 25 to 75 mL/min/1.73 m^2^ and an UACR of 200 to 5000 mg/g with or without DM. Most patients were treated with ACE-inhibitors (31.5%) or angiotensin II type 1 receptor blockers (ARB in 66.7%), and 67.5% had DM. The mean eGFR and albuminuria were 43 mL/min/1.73 m^2^ and 949 mg/g, respectively. After 2.4 years, the primary composite renal endpoint (sustained decline of eGFR of at least 50%, end-stage kidney disease, or renal or CV death) was significantly reduced in the dapagliflozin group compared to placebo (HR 0.61, 95% CI 0.51 to 0.72) with a number-needed-to treat of 19. The primary endpoint was mostly driven by a reduction in eGFR decline and progression to end-stage kidney disease [79]. Regarding secondary endpoints, a combined endpoint of HHF and CV death was significantly reduced (HR 0.71, 95% CI 0.55 to 0.92), as was all-cause mortality (HR 0.69, 95% CI 0.53 to 0.88). Furthermore, a primary pre-specified outcome “abrupt decline in kidney function”, indicated by a doubling of serum creatinine, was reduced in the dapagliflozin group compared to placebo (HR 0.68, 95% CI 0.49 to 0.94). This effect was consistent independently of baseline eGFR, diuretic medication, type 2 DM, or heart failure [80].

For the first time, subgroup analyses of DAPA-CKD showed a consistent effect of dapagliflozin also in individuals with CKD but without DM for the primary renal endpoint (HR 0.5, 95% CI 0.35 to 0.72), which was later confirmed with empagliflozin in EMPA-KIDNEY, too. In a subgroup of DAPA-CKD consisting of 270 individuals with IgA-nephropathy, 254 of whom were diagnosed by biopsy, dapagliflozin showed a significant reduction of the primary composite renal endpoint (HR 0.29, 95% CI 0.12 to 0.73). Therefore, dapagliflozin, besides RAS-inhibition, might be used for the treatment of IgA-nephropathy [57].

#### 8.11.4. Canagliflozin

##### Canagliflozin in Diabetes Mellitus

The CANVAS-program, including the CANVAS and CANVAS-renal trials in 10,142 individuals with DM and CV disease or high CV risk, showed a reduction of a secondary efficiency composite renal endpoint, consisting of a sustained 40% reduction in eGFR, the need for renal replacement therapy, or renal death, for canagliflozin compared to placebo (HR 0.60, 95% CI 0.47 to 0.77). In CANVAS, the mean eGFR was 76.5 mL/min/1.73 m^2^ and 20.7% of patients had an eGFR of <60 mL/min/1.73 m^2^ [55].

##### Canagliflozin in Chronic Kidney Disease

The CREDENCE-trial was the first study with a primary cardio-renal composite endpoint and randomized 4401 people with DM and CKD with an eGFR of ≥30 to <90 mL/min/1.73 m^2^ and macroalbuminuria of >300 to ≤5000 mg/g to receive either canagliflozin or placebo as an add-on to the standard of care. The mean eGFR was 56.2 ml/min/1.73 m^2^, and the mean albuminuria was 923 mg/g. Due to efficacy, the trial was terminated early after a planned interim analysis. Canagliflozin demonstrated a reduction of the composite renal endpoint, consisting of end-stage renal disease (dialysis, transplantation, sustained eGFR of <15 mL/min/1.73 m^2^), a doubling of the serum creatinine level, or renal or CV death (HR 0.70, 95% CI 0.59 to 0.82) [56]. A key secondary CV outcome containing HHF and CV death was significantly reduced as well (HR 0.69, 95% CI 0.57 to 0.83).

#### 8.11.5. Ertugliflozin

##### Ertugliflozin in Diabetes Mellitus

The VERTIS CV-trial randomized people with DM type 2 and established atherosclerotic CV disease involving the coronary, cerebrovascular, or peripheral arterial systems to receive ertugliflozin or placebo. A secondary kidney composite endpoint including a doubling of serum creatinine, dialysis, kidney transplantation, or renal death showed no statistically significant difference (HR 0.81; 95% CI 0.63 to 1.04). Secondary explanatory analyses demonstrated a slower decline in eGFR (2.6 mL/min/1.73 m^2^; 95% CI 1.5 to 3.6) and reduced albuminuria (−16.2%; 95% CI −23.9% to −7.6%) with ertugliflozin compared to placebo at 60 months [81,82].

##### Meta-Analyses of Renal Effects of SGLT2-I

A meta-analysis of EMPA-REG OUTCOME, the CANVAS program, CREDENCE, and DECLARE-TIMI 58 showed a significant relative risk reduction of a composite kidney endpoint including dialysis, transplantation, or renal death (relative risk 0.67, 95% CI 0.52 to 0.86) with consistent effects across all studies. Therapy with SGLT2-I was beneficial in all eGFR-subgroups, including patients with reduced eGFR between 30 and 45 mL/min/1.73 m^2^ [83]. Furthermore, a meta-analysis recently published confirmed a significant relative risk reduction for kidney disease progression with SGLT2-I compared to placebo (relative risk 0.63, 95% CI 0.58 to 0.69) independently of DM type 2 [84].

##### Adverse Outcomes

Individuals with DM and SGLT2-I showed an increased risk of genital infections, atypical ketoacidosis, and Fournier’s gangrene. According to the European Medical Association (EMA), urinary tract infections, genital infections, and polyuria are all common side effects. The rate of these side effects ranges, according to the studied patient collective (e.g., patients with or without diabetes), from ≥1/100 to <1/10. Atypical ketoacidosis (ca. 5 of 1000) and Fournier-gangrene (1.6 of 100,000) are rare or very rare, respectively [85].

## 9. Closing Remarks and Conclusions

Today, numerous glucose-lowering drugs for the treatment of individuals with DM are available. An intensive glucose-lowering therapy using insulin, sulfonylureas, or metformin demonstrated a beneficial effect in reducing micro- and macroalbuminuria by improving glycemic control, and therefore, an HbA1c-focused approach has been established until recently [9]. However, a significant reduction in CV or renal endpoints solely due to improved glycemic control could not be demonstrated. On the contrary, the ACCORD trial showed increased mortality with intensive therapy targeting normal HbA1c-levels [11].

In order to reduce CV and renal events, for many years glucose-lowering has been embedded in a comprehensive strategy for people with DM, especially in individuals with DM and CV or renal risk [86]. Lifestyle modifications, including smoking cessation, increased physical activity, weight control, and dietary counseling, are the foundation of this approach. Furthermore, blood pressure control in hypertensive individuals, preferably with an angiotensin converting enzyme inhibitor or an angiotensin II_1_ receptor antagonist at a maximally tolerable dose, can reduce micro- and macro-albuminuria, prevent the progression of diabetic kidney disease and reduce mortality [87]. Beyond that, the INNOVATION trial demonstrated a reduction in albuminuria in individuals with DM without hypertension, too [88]. Moreover, statin treatment should be considered for primary prevention in individuals at high CV risk [89,90].

Implementation of this comprehensive strategy with the attainment of improved glycemic control, use of ACE- or AT II_1_-blockers (RAS-blockade), and statin treatment reduced the risk of nephropathy, progression to end-stage kidney disease, and CV mortality in the STENO-2 trial [91,92].

With the introduction of SGLT-2-inhibitors and GLP-1-RA, new glucose-lowering drugs for individuals with DM at high CV or renal risk are available. GLP-1-RAs showed effective glycemic control, improved blood pressure, and reduced progression to macroalbuminuria, but were not able to delay the progression of renal disease [41]. Finally, SGLT-2-inhibitors demonstrated convincing evidence in slowing the progression of renal and CV disease in individuals with DM, which has been extensively outlined above. Especially remarkable is the nephroprotective effect of SGLT-2-inhibition beyond glycemic control. Based on this evidence, the 2022 update of the KDIGO guidelines for diabetes management in chronic kidney disease recommend the initiation of treatment with an SGLT-2-inhibitor in people with DM and CKD having an eGFR of at least ≥20 mL/min/1.73 m^2^ [86]. By this, SGLT-2-inhibition has been established as a first-line treatment besides metformin (only if eGFR is >30 mL/min/1.73 m^2^), RAS-inhibition, and statin-therapy in individuals with DM at renal risk. Furthermore, a shift from an HbA1c-focused approach to therapy with drugs with the verifiable improvement of CV- and renal endpoints has been initiated. As SGLT-2 inhibitors also showed nephroprotective properties in individuals without DM, a glycemia-independent nephroprotective potential is plausible.

## Figures and Tables

**Table 1:** Overview of cardiovascular and renal outcome trials showing positive effects of SGLT-2-inhibitors.

	EMPA-REG Outcome	EMPA-KIDNEY	CANVAS-Program	CREDENCE	DECLARE-TIMI 58	DAPA-CKD
Drug	empagliflozin (10 mg or 25 mg)	empagliflozin (10 mg)	canagliflozin (100 mg or 300 mg)	canagliflozin (100 mg)	dapagliflozin (10 mg)	Dapagliflozin (10 mg)
Total of participants (n)	7.020	6.609	10.142	4.401	17.160	4.304
CVD (%)	100	27	66	50.4	41	37.4
Heart failure (%)	10.1	9.9	14.4	14.8	10	10.9
Chronic kidney disease (%)		100		100		
Follow up (years)	3.1	2.0	3.6	2.6	4.2	2.4
Kidney outcome or composite kidney outcome	incident or worsening of nephropathy (progression to macroalbuminuria *, doubling of serum-creatinine, initiation of renal replacement therapy, or renal death	progression of kidney disease (end-stage kidney disease, a sustained decrease in eGFR to <10 mL/min/1.73 m^2^, a sustained decrease in eGFR of ≥40% from baseline, or death from renal causes) or death from cardiovascular causes	Composite doubling in serum creatinine, kidney failure, or death from kidney causes	Composite of kidney failure, doubling of serum creatinine, or death from kidney or CV causes	Composite of ≥40% decrease in eGFR to <60 mL/min/1.73 m^2^, kidney failure, CV or renal death	Composite of sustained decline in eGFR to <10 mL/min/1.73 m^2^, sustained decline in eGFR ≥40%, or renal or CV death
Kidney outcome result	12.7% vs. 18.8% HR 0.61 (0.53; 0.7)	13.1% vs. 16.9% HR 0.72 (0.64; 0.82)	5.5% vs. 9.0% (per 1000 patient years) HR 0.60 (0.47; 0.77)	11.1% vs. 15.5% HR 0.70 (0.59; 0.82)	4.3% vs. 5.6% HR 0.76 (0.67; 0.87)	9.2% vs. 14.5% HR 0.61 (0.51; 0.72)
Number needed to treat	17	26	286 (per year)	23	77	19

* only individuals without baseline macroalbuminuria analysed; CVD = cardiovascular disease; HR = Hazard Ratio; CV = cardiovascular.

## Data Availability

Not applicable.
